# Incidence, Mortality Features and Lifetime Risk Estimation of Digestive Tract Cancers in an Urban District of Shanghai, China

**DOI:** 10.1007/s44197-022-00047-3

**Published:** 2022-06-25

**Authors:** Jing-Hao Bi, Hui-Yun Yuan, Yu Jiang, Yun Zhang, Wen-Wei Zheng, Lei Zhang, Zhuo-Ying Li, Hong-Lan Li, Yu-Ting Tan, Wen-Sui Zhao, Yong-Bing Xiang

**Affiliations:** 1 School of Public Health, Shanghai Jiaotong University School of Medicine, Shanghai, 200025 China; 2 Renji Hospital, Shanghai Jiaotong University School of Medicine, Shanghai, 200127 China; 3 State Key Laboratory of Oncogene and Related Genes & Department of Epidemiology, Shanghai Cancer Institute, Renji Hospital, Shanghai Jiaotong University School of Medicine, No. 25, Lane 2200, Xie Tu Road, Shanghai, 200032 People’s Republic of China; 4 Shanghai Changning District Center for Disease Control and Prevention, No. 39, Yun Wu Shan Road, Shanghai, 200051 People’s Republic of China

**Keywords:** Digestive tract cancers, Incidence, Mortality, Lifetime risk, Shanghai

## Abstract

Digestive tract cancers are the common cause of cancer deaths in both China and worldwide. This study aimed to describe the burden, recent trends and lifetime risks in the incidence and mortality of digestive tract cancers in an urban district of Shanghai, China. Our study extracted data on stomach, colon, rectum and liver cancers diagnosed in Changning District between 2010 and 2019 from the Shanghai Cancer Registry. We calculated age-standardized incidence and mortality rates, the risks of developing and dying from cancer, and the estimated annual percent changes. Between 2010 and 2019, 8619 new cases and 5775 deaths were registered with digestive tract cancers in the district. The age-standardized incidence rates (ASIRs) of liver cancer decreased steadily, whereas the ASIRs of stomach, colon and rectum cancers remained stable from 2010 to 2019. The age-standardized mortality rates (ASMRs) of stomach and liver cancers showed significant declining changes from 2010 to 2019 in both sexes, but that of colon and rectum cancers remained stable during the entire period. The risks of developing and dying from digestive tract cancers were substantially higher in men than women. The burden of digestive tract cancer and its disparities between sex and age group remain major public health challenges in urban Shanghai. To reduce the burden of digestive tract cancers, the government and researchers should develop and promote a healthy diet, organize a screening, and reduce the prevalence of smoking, alcohol drinking, and hepatitis B virus and hepatitis C virus infections.

**Supplementary Information:**

The online version contains supplementary material available at 10.1007/s44197-022-00047-3.

## Introduction

Stomach, colon, rectum and liver cancers are the four most common types of digestive tract cancer, which is one of the most commonly diagnosed malignancies and one of the leading causes of cancer-related death worldwide, particularly in China [1, 2]. It was estimated that 19,292,789 new cancer cases and 9,958,133 cancer deaths occurred worldwide in 2020, among which 20.09% of new cases and 25.25% of deaths could be attributed to digestive tract cancers [1]. According to the cancer statistics of the National Cancer Center (NCC) of China in 2016, stomach cancer ranked the third highest incidence and the third most common cause of death, with an estimated age-standardized incidence rate (ASIR) was 17.59/10^5^ and an estimated age-standardized mortality rate (ASMR) was 12.30/10^5^. Colorectal cancer had the second incidence and fourth mortality, the estimated ASIR and ASMR were 18.05/10^5^ and 8.12/10^5^, respectively. Liver cancer is the fourth most common cancer and the second in cancer death, with ASIR and ASMR reaching as high as 17.65/10^5^ and 15.07/10^5^, respectively [3].

Epidemiological evidence indicated the occurrence and development of digestive tract cancers are related to smoking, alcohol drinking, unhealthy diet, obesity, physical inactivity, virus infection, and others [4–7]. Shanghai is the largest metropolis, with ongoing changes in lifestyle and diet, diverse cultures, and a prosperous economy in China. With the accelerating urbanization process, the proportion of high-fat, high-protein and low-fiber diets is increasing, and there might be new changes in the incidence and mortality of digestive tract cancers [8–10]. Therefore, we analyzed the burden, features, and recent trends in the incidence and mortality rates of stomach, colon, rectum and liver cancers from 2010 to 2019 in an urban district of Shanghai, as well as the lifetime risks estimation, to provide evidence of local disease burden and to optimize cancer control strategy in terms of public health.

## Materials and Methods

### Data and Design

The high-quality data of this study was based on the Shanghai Cancer Registry, which has a standardized cancer-reporting procedure. The data of the Shanghai Cancer Registry has been published in the volumes of the Cancer Incidence in Five Continents of the International Agency for Research on Cancer (IARC) [11]. The Changning District, located in the west of Shanghai, has a total permanent and median size population of 626,000. It is one of the original urban districts with less resident mobility than other suburb districts. The cancer data for this region were primarily collected as a part of the Shanghai Cancer Registry by the Shanghai Changning District Center for Disease Control and Prevention using a standardized cancer reporting system [12]. No identity information of the individuals was included in our data analysis. Cancer cases and deaths were coded using the International Classification of Diseases, the tenth revision (ICD-10). Stomach cancer (C16), colon cancer (C18), rectum cancer (C19–20), and liver cancer (C22) were included in our current analysis.

Except for the population census year 2010, population data (age composition by sex) were obtained from the Changning District Bureau of Public Security. The gender and age group population data were estimated using the interpolation or extrapolation method [13] during the remaining non-census years. The study was approved by the Renji Hospital Ethics Committee of Shanghai Jiao Tong University School of Medicine (KY2019-197, KY2021-028).

### Data Quality Control

The quality of registration data was assessed based on the criteria of the “Guideline for Chinese Cancer Registration” [14]. Briefly, the completeness, validity, reliability, and comparability of the data were evaluated using a series of standards, including the mortality to incidence ratio (M/I) and the basis of diagnosis with the highest reliability, that is, percentage of histologically verified (HV%), biochemistry, surgery, imaging, clinical, and death certification only (DCO%). The corresponding quality control indicators of the data for digestive tract cancers in our study were 0.67, 72.77%, 8.02%, 7.80%, 8.83%, 2.52% and 0.07%, respectively (Supplemental Table 1). These indicators suggested that the overall quality of data was satisfied.

### Statistical Analysis

The crude incidence and mortality rates of digestive tract cancers were calculated and shown as per 100 000 (10^5^) person-years. The direct standardization method with Segi’s world standard population [15] was used for age-standardized rates. Lifetime risk was defined as the probability of developing or dying from digestive tract cancers from birth to 85 years old, calculated using the DevCan software, version 6.7.9 [16, 17]. The Joinpoint Regression Program version 4.9.0.0 was used to calculate the annual percent change (APC) in annual trends of ASIRs and ASMRs, and the *Z* test was employed to assess whether the APC was statistically different from zero [18]. All models were restricted to a maximum of 2 joinpoints (3 line segments) [19]. Age-specific incidence and mortality rates were calculated for each 5-year age group (0–4 to 85+).

## Results

### Incidence Analysis

As shown in Table 1, a total of 2554 stomach cancer cases, 2922 colon cancer cases, 1625 rectum cancer cases, and 1518 liver cancer cases were registered from 2010 to 2019 in Changning district, Shanghai. The corresponding crude incidence rates of the four sites were 42.55/10^5^,48.68/10^5^, 27.07/10^5^, and 25.29/10^5^, respectively. After using the Segi’s world standard population, the ASIRs of digestive tract cancers were higher in males than females. The ratio of male to female cases was 1.79:1 for stomach cancer, 1.14:1 for colon cancer, 1.52:1 for rectum cancer, and 2.39:1 for liver cancer, respectively. The most common type of digestive tract cancer was stomach cancer in men (21.51/10^5^) and colon cancer in women (16.66/10^5^) from 2010 to 2019 in the district. The ASIRs of stomach, colon, rectum and liver cancer were 16.60/10^5^, 19.16/10^5^, 11.19/10^5^, and 11.46/10^5^ during 2010–2004, and decreased to 15.99/10^5^, 18.06/10^5^, 10.70/10^5^, and 8.93/10^5^, respectively, during 2015–2019 (Table 1).

**Table 1:** Crude and age-standardized incidence rates of digestive tract cancers in the Changning District, Shanghai, China, 2010–2019

Sites	Years	Male			Female			Total		
		Number	CR	ASIR	Number	CR	ASIR	Number	CR	ASIR
Stomach	2010–2014	789	51.48	21.52	451	28.93	11.98	1240	40.11	16.60
	2015–2019	848	59.91	21.32	466	31.15	11.01	1314	45.13	15.99
	Total	1637	55.53	21.51	917	30.02	11.46	2554	42.55	16.33
Colon	2010–2014	734	47.89	20.71	682	43.76	17.78	1416	45.81	19.16
	2015–2019	820	57.93	20.35	686	45.85	15.95	1506	51.72	18.06
	Total	1554	52.71	20.34	1368	44.78	16.66	2922	48.68	18.41
Rectum	2010–2014	470	30.67	13.78	322	20.66	8.70	792	25.62	11.19
	2015–2019	509	35.96	13.43	324	21.66	8.08	833	28.61	10.70
	Total	979	33.21	13.61	646	21.15	8.29	1625	27.07	10.90
Liver	2010–2014	560	36.54	17.53	243	15.59	5.43	803	25.98	11.46
	2015–2019	510	36.03	13.78	205	13.70	4.33	715	24.56	8.93
	Total	1070	36.29	15.71	448	14.67	4.86	1518	25.29	10.20

All rates in this table are shown as per 100,000 persons

*CR* crude rate, *ASIR* age-standardized incidence rate, taking standard world population of Segi’s (1960) as the standard

### Mortality Analysis

Between 2010 and 2019, 1 865 stomach cancer deaths, 1 643 colon cancer deaths, 935 rectum cancer deaths, and 1 332 liver cancer deaths were reported in the Changning District, as presented in Table 3. The corresponding crude mortality rates of digestive tract cancer were 31.07/10^5^, 27.37/10^5^, 15.58/10^5^, and 22.19/10^5^, respectively. The lowest ASMR observed in female rectum cancer was 2.72/10^5^ (2015–2019) and the highest in male stomach cancer was 15.36/10^5^ (2010–2014). The ratios of male to female deaths for stomach, colon, rectal and liver cancers were 1.96:1, 1.13:1, 1.58:1, and 2.48:1, respectively, which was paired with one of the new cases. In addition, the trend for the crude mortality rates is not entirely consistent with that of ASMRs. For example, the crude mortality trend of colon cancer increased from 2010–2014 to 2015–2019. While the ASMRs of that decreased slightly from 2010–2014 to 2015–2019 (Table 2).

**Table 2:** Crude and age-standardized mortality rates of digestive tract cancers in the Changning District, Shanghai, China, 2010–2019

Sites	Years	Male			Female			Total		
		Number	CR	ASMR	Number	CR	ASMR	Number	CR	ASMR
Stomach	2010–2014	647	42.22	15.36	335	21.49	7.21	982	31.77	11.01
	2015–2019	588	41.54	11.78	295	19.72	5.02	883	30.33	8.18
	Total	1235	41.89	13.47	630	20.62	6.13	1865	31.07	9.56
Colon	2010–2014	384	25.06	9.13	376	24.12	7.47	760	24.59	8.23
	2015–2019	486	34.33	9.54	397	26.54	6.27	883	30.33	7.80
	Total	870	29.51	9.40	773	25.31	6.81	1643	27.37	8.01
Rectum	2010–2014	258	16.83	6.08	181	11.61	4.06	439	14.20	4.99
	2015–2019	314	22.18	6.55	182	12.17	2.72	496	17.04	4.56
	Total	572	19.40	6.35	363	11.88	3.37	935	15.58	4.78
Liver	2010–2014	500	32.62	15.18	193	12.38	3.87	693	22.42	9.48
	2015–2019	449	31.72	11.77	190	12.70	3.55	639	21.95	7.56
	Total	949	32.19	13.37	383	12.54	3.72	1332	22.19	8.46

All rates in this table are shown as per 100,000 persons

*CR* crude rate, *ASMR* age-standardized mortality rate, taking standard world population of Segi’s (1960) as the standard

### Lifetime and Age-Conditional Risks Estimation

The lifetime risks of developing stomach cancer were estimated to be 4.80% in men and 2.37% in women (Table 3). The lifetime risks of dying from stomach cancer were estimated to be 3.74% in men and 1.51% in women (Table 4). These probabilities mean that one in twenty-one males and one in forty-two females will develop stomach cancer from birth to 85 years old, and one in forty-seven males and one in sixty-six females will die of stomach cancer.

**Table 3:** Probabilities of developing digestive tract cancers by site and sex in the Changning District, Shanghai, China, 2010–2019^a^

Sites	Sex	Birth to 49	50–59	60–69	70–85	Birth to 85
Stomach	Male	0.18 (1 in 556)	0.46 (1 in 217)	1.09 (1 in 92)	3.13 (1 in 32)	4.80 (1 in 21)
	Female	0.17 (1 in 588)	0.28 (1 in 357)	0.48 (1 in 208)	1.46 (1 in 68)	2.37 (1 in 42)
Colon	Male	0.16 (1 in 625)	0.37 (1 in 270)	1.09 (1 in 92)	3.16 (1 in 32)	4.72 (1 in 21)
	Female	0.16 (1 in 625)	0.36 (1 in 278)	0.83 (1 in 120)	2.35 (1 in 43)	3.67 (1 in 27)
Rectum	Male	0.14 (1 in 714)	0.35 (1 in 286)	0.69 (1 in 145)	1.65 (1 in 61)	2.80 (1 in 36)
	Female	0.13 (1 in 769)	0.21 (1 in 476)	0.36 (1 in 278)	1.00 (1 in 100)	1.70 (1 in 59)
Liver	Male	0.27 (1 in 370)	0.50 (1 in 200)	0.66 (1 in 152)	1.36 (1 in 74)	2.76 (1 in 36)
	Female	0.05 (1 in 2000)	0.09 (1 in 1111)	0.20 (1 in 500)	0.86 (1 in 116)	1.19 (1 in 84)

^a^ For people free of cancer at beginning of age interval

**Table 4:** Probabilities of dying from digestive tract cancers by site and sex in the Changning District, Shanghai, China, 2010–2019^a^

Sites	Sex	Birth to 49	50–59	60–69	70–85	Birth to 85
Stomach	Male	0.06 (1 in 1667)	0.22 (1 in 455)	0.53 (1 in 189)	2.96 (1 in 34)	3.74 (1 in 27)
	Female	0.05 (1 in 2000)	0.14 (1 in 714)	0.22 (1 in 455)	1.11 (1 in 90)	1.51 (1 in 66)
Colon	Male	0.04 (1 in 2500)	0.10 (1 in 1000)	0.38 (1 in 263)	2.04 (1 in 49)	2.55 (1 in 39)
	Female	0.04 (1 in 2500)	0.10 (1 in 1000)	0.23 (1 in 435)	1.49 (1 in 67)	1.85 (1 in 54)
Rectum	Male	0.03 (1 in 3333)	0.10 (1 in 1000)	0.26 (1 in 385)	1.36 (1 in 74)	1.74 (1 in 57)
	Female	0.02 (1 in 5000)	0.06 (1 in 1667)	0.11 (1 in 909)	0.65 (1 in 154)	0.85 (1 in 118)
Liver	Male	0.19 (1 in 526)	0.40 (1 in 250)	0.59 (1 in 169)	1.36 (1 in 74)	2.51 (1 in 40)
	Female	0.03 (1 in 3333)	0.06 (1 in 1667)	0.14 (1 in 714)	0.76 (1 in 132)	0.99 (1 in 101)

^a^ For people free of cancer at beginning of age interval

Between birth to 85 years old, the lifetime risks of developing cancer in men were highest for the stomach (4.80%), followed by the colon (4.72%), rectum (2.80%) and liver (2.76%), and it was highest for colon (3.67%), followed by stomach (2.37%), rectum (1.70%) and liver (1.19%) in women, respectively (Table 3). Before the age of 50, the cancer site with the highest age-conditional developing risk was the liver for males and the stomach for females. The age-conditional risk of developing colon cancer was the highest for older people between the ages of 60 and 85 regardless of gender (Table 3).

Between birth to 85 years old, the lifetime risk of dying from cancer in men was highest for the stomach (3.74%), followed by the colon (2.55%), liver (2.51%) and rectum (1.74%), and was highest for colon (1.85%), followed by stomach (1.51%), liver (0.99%) and rectum (0.85%) in women, respectively (Table 4). Before the age of 60, the sites with the highest age-conditional dying risks were liver cancer in men and stomach cancer in women. Between the ages of 60 and 69, the sites with the highest age-conditional dying risks were liver cancer in men and colon cancer in women. Between the ages of 70 and 85, the sites with the highest age-conditional dying risks were stomach cancer in men and colon cancer in women (Table 4).

### Joinpoint Regression Analysis

As shown in Table 5, for both sexes combined, the total ASIRs of stomach, colon, and rectum cancers remained stable, but that of liver cancer significantly decreased by 5.18% per year from 2010 to 2019. The time trends of ASIRs in men were similar to the total trends over the whole period. In women, the ASIRs for cancers of the colon, rectum, and liver showed no changes in time trends. The time trends of ASIRs for stomach cancer in women remarkably decreased from 2010 to 2013, but remained stable from 2013 to 2019 (Table 5).

**Table 5:** Joinpoint regression analysis of age-standardized incidence of digestive tract cancers in the Changning District, Shanghai, China, 2010–2019

Sites	Sex	AAPC (%, 95% CI)	Trend 1		Trend 2	
			Years	APC (%, 95%CI)	Years	APC (%, 95%CI)
Stomach	Male	− 1.12 (− 4.54, 2.43)	2010–2019	− 1.12 (− 4.54, 2.43)		
	Female	− 2.97 (− 7.21, 1.46)	2010–2013	− 15.98* (− 27.07, − 3.20)	2013–2019	4.27 (− 1.01, 9.83)
	Total	− 1.44 (− 4.5, 1.72)	2010–2019	− 1.44 (− 4.5, 1.72)		
Colon	Male	1.22 (− 3.42, 6.08)	2010–2019	1.22 (− 3.42, 6.08)		
	Female	− 1.71 (− 5.7, 2.44)	2010–2019	− 1.71 (− 5.7, 2.44)		
	Total	− 0.13 (− 4.22, 4.14)	2010–2019	− 0.13 (− 4.22, 4.14)		
Rectum	Male	1.11 (− 2.62, 4.99)	2010–2019	1.11 (− 2.62, 4.99)		
	Female	− 2.49 (− 6.43, 1.61)	2010–2019	− 2.49 (− 6.43, 1.61)		
	Total	− 0.26 (− 2.77, 2.31)	2010–2019	− 0.26 (− 2.77, 2.31)		
Liver	Male	− 5.58* (− 10, − 0.9)	2010–2019	− 5.58* (− 10, − 0.9)		
	Female	− 2.69 (− 7.3, 2.14)	2010–2019	− 2.69 (− 7.3, 2.14)		
	Total	− 5.18* (− 8.4, − 1.8)	2010–2019	− 5.18* (− 8.4, − 1.8)		

*AAPC* average annual percent change, *APC* annual percent change, *CI* confidence interval

*P < 0.05

For both sexes combined, the total ASMRs of colon and rectum cancers remained stable, but that of stomach and liver cancers decreased from 2010 to 2019. In men, the ASMRs had decreased for cancers of the stomach and liver, whereas stable trends were detected for cancers of the colon and rectum. In women, the mortality rates were relatively stable for colon and liver cancers. For cancers of the stomach and rectum in women, declining trends in ASMRs were observed during the whole period (Table 6).

**Table 6:** Joinpoint regression analysis of age-standardized mortality of digestive tract cancers in the Changning District, Shanghai, China, 2010–2019

Types	Sex	AAPC (%, 95%CI)	Trend 1	
			Years	APC (%, 95%CI)
Stomach	Male	− 4.58* (− 7.52, − 1.54)	2010–2019	− 4.58* (− 7.52, − 1.54)
	Female	− 5.68* (− 10.63, − 0.46)	2010–2019	− 5.68* (− 10.63, − 0.46)
	Total	− 4.93* (− 8.07, − 1.69)	2010–2019	− 4.93* (− 8.07, − 1.69)
Colon	Male	0.22 (− 3.68, 4.29)	2010–2019	0.22 (− 3.68, 4.29)
	Female	− 3.25 (− 7.38, 1.06)	2010–2019	− 3.25 (− 7.38, 1.06)
	Total	− 1.37 (− 4.48, 1.84)	2010–2019	− 1.37 (− 4.48, 1.84)
Rectum	Male	1.72 (− 1.45, 4.99)	2010–2019	1.72 (− 1.45, 4.99)
	Female	− 8.57* (− 13.35, − 3.53)	2010–2019	− 8.57* (− 13.35, − 3.53)
	Total	− 1.99 (− 4.2, 0.28)	2010–2019	− 1.99 (− 4.2, 0.28)
Liver	Male	− 5.89* (− 9.99, − 1.6)	2010–2019	− 5.89* (− 9.99, − 1.6)
	Female	− 2.2 (− 7.3, 3.19)	2010–2019	− 2.2 (− 7.3, 3.19)
	Total	− 5.11* (− 8.1, − 2.03)	2010–2019	− 5.11* (− 8.1, − 2.03)

*AAPC* average annual percent change, *APC* annual percent change, *CI* confidence interval

*P < 0.05

### Age-Specific Incidence and Mortality Rates

**Fig. 1:**
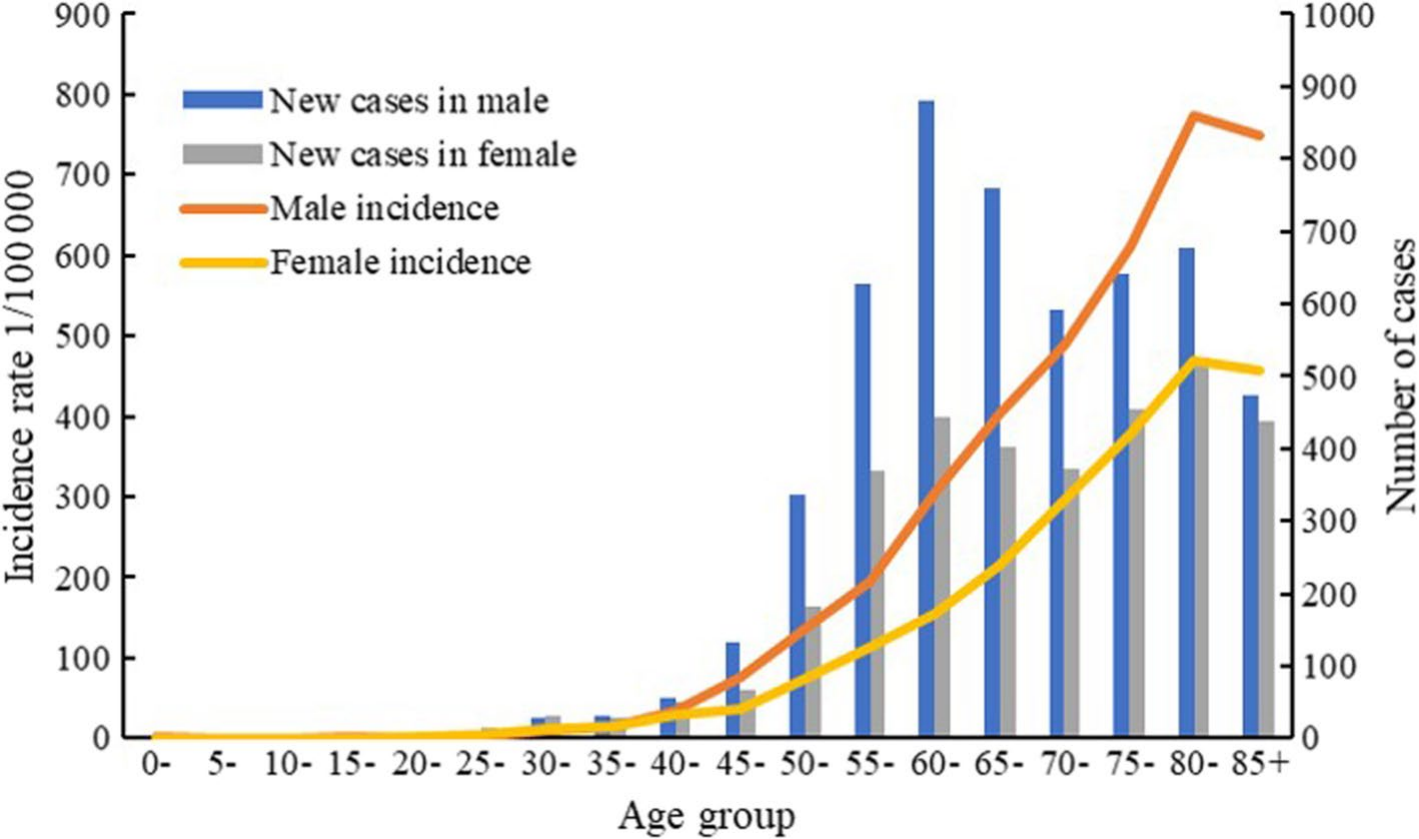
Age-specific incidence rates and numbers of new cases of digestive tract cancers in the Changning District, Shanghai, China, 2010–2019

The age-specific incidence rate was relatively low before 40 years old, and increased dramatically since then, reaching a peak at the age group of 80–84 years both in men and women (Fig. 1). The number of cases increased with age before 65 years old both in men and women (Fig. 1). The highest number of cases occurred between the age group of 60–64 years in men, while between the age group of 80–84 years in women (Fig. 1). After the age group of 40–44 years, incidence rates were substantially higher in men than in women. Supplementary Tables 2 and 3 show the Joinpoint regression analysis results of incidence trends of digestive tract cancers by age groups in men and women.

**Fig. 2:**
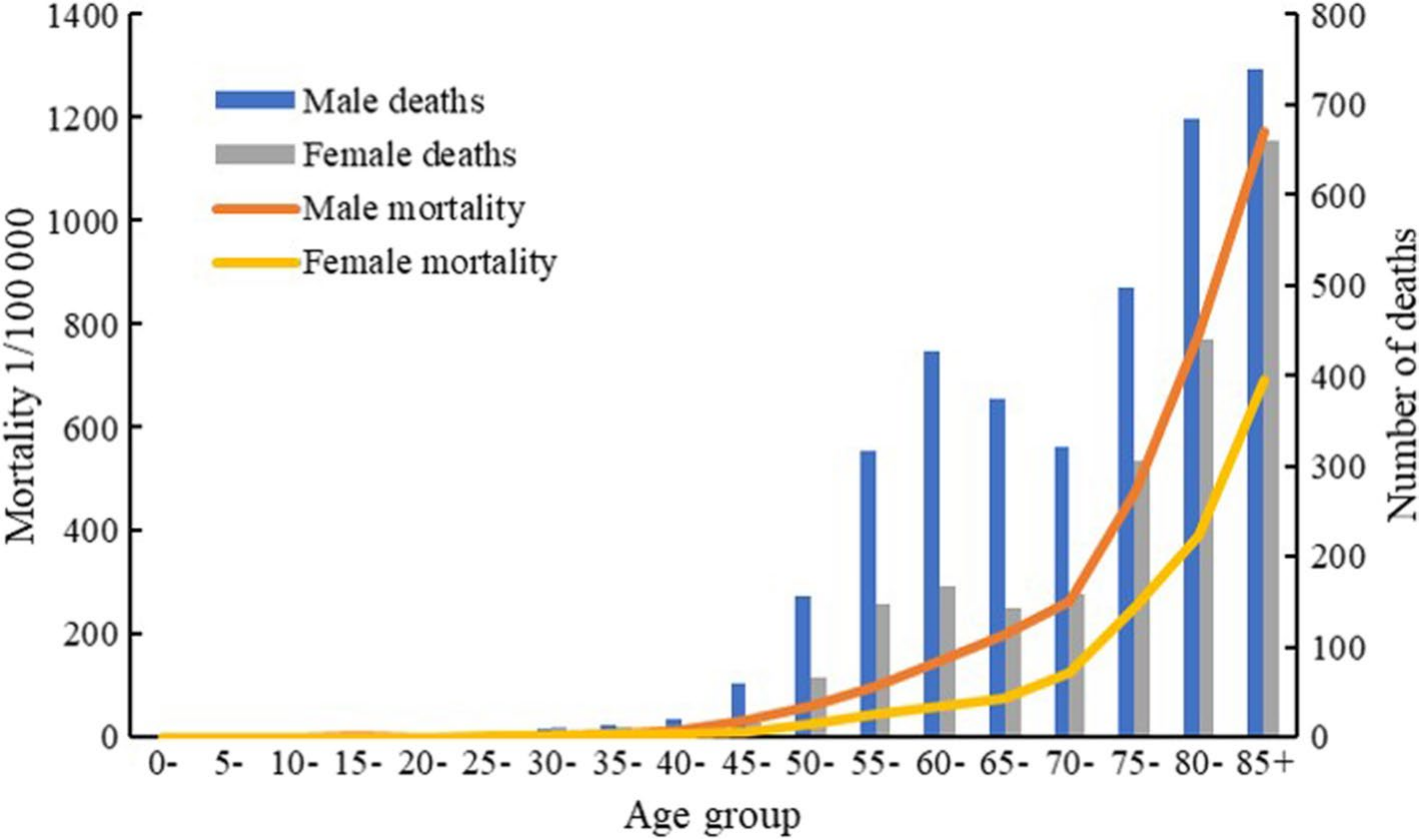
Age-specific mortality rates and numbers of deaths of digestive tract cancers in the Changning District, Shanghai, China, 2010–2019

The age-specific mortality rates were relatively low before 50 years, then gradually increased in the age group of 50–70 years, dramatically increased after 70 years, and reached a peak after 85 years (Fig. 2). In all age groups, the mortality rates of digestive tract cancers were consistently higher in men than women (Fig. 2). The highest number of cancer deaths occurred in the age group of 85+ in both men and women (Fig. 2). Supplementary Tables 4 and 5 show the results of Joinpoint regression analysis of mortality trends of digestive tract cancers by age group in men and women.

## Discussion

Based on a high-quality population-based of cancer incidence and mortality data, we conducted a comprehensive statistical analysis of digestive tract cancers in an urban district of Shanghai, a higher developing economy and society in China. In this study, we first briefly described the features of incidence and mortality for primary digestive tract cancers (stomach, colon, rectal, and liver cancers) in the Changning District, Shanghai from 2010 to 2019. Secondly, we calculated the risks of developing and dying from digestive tract cancers during certain age intervals to reflect life expectancy and the risk during their lifetime. Thirdly, we used the Joinpoint regression analysis to investigate the incidence and mortality trends of digestive tract cancers. Finally, we presented the number of new cases and deaths in all age groups, as well as the recent trends of age-specific rates by sex during the period 2010–2019.

Stomach cancer is the most common digestive tract cancer in China with a poor prognosis. The age-standardized 5-year relative survival rate was 35.1% from 2012 to 2015 [20]. It has been reported that factors such as *Helicobacter pylori* infection, smoking, alcohol drinking, obesity, high-salt diet, low fruit intake, and the high consumption of processed meat and grilled or barbecued meat and fish are related to the risk of stomach cancer in China [1, 21, 22]. Based on our data from urban Shanghai, the ASIRs of stomach cancer incidence of 21.32/10^5^ in men and 11.01/10^5^ in women during 2015–2019, which were about 1.4-fold higher than the global average in 2020 (men: 15.8/10^5^; women: 7.0/10^5^ in women) [1], but they are lower than the Chinese average in 2019 (men: 47.4/10^5^; women: 15.8/10^5^) [23]. The ASMRs of stomach cancer were 11.78/10^5^ in men and 5.05/10^5^ in women from 2015 to 2019, which was slightly higher than the global average in 2020 (men: 11.0/10^5^; women: 4.9/10^5^) [1], but lower than the Chinese average in 2019 (men: 33.1/10^5^; women: 12.2/10^5^) [23].

A stable trend was observed for the ASIRs of stomach cancer from 2010 to 2019, whereas the crude rate of stomach increased, which was consistent with our previous study [24]. This is because of changes in the population age structure and the risk factors such as high-salt foods, obesity and others [25]. Similar to other countries or regions [7, 26], the ASMRs of stomach cancer decreased by 4.93% annually from 2010 to 2019. This might be attributed to adequate medical resources, higher levels of health care, improvements in early diagnosis, etc. The incidence and mortality of stomach cancer are 1.7-fold and 1.9-fold higher in men than in women, consistent with a previous study in China [27]. This difference may be related to the different gender levels of the proportion of exposure factors such as smoking and alcohol drinking [28–30].

Colorectal cancer can be considered a marker of socioeconomic development and, in countries undergoing a major transition, incidence rates tend to rise uniformly with increasing human development index [31, 32]. Shanghai, one of the representatives of China's eastern coastal cities, has a high economic and social development level. The incidence and mortality of colorectal cancer almost reach those in developed countries. Compared with GLOBOCAN 2020 data [1, 33], the ASIRs of colorectal cancer for both sexes combined in the Changning District of Shanghai from 2015 to 2019 (28.76/10^5^) were much higher than the global average (19.5/10^5^) and the China average (23.9/10^5^). When it comes to the sex and type of colorectal cancer, the ASMRs of colon cancer and rectum cancer in our study from 2015 to 2019 (colon cancer in men: 9.54/10^5^; colon cancer in women: 6.27/10^5^; rectum cancer in men: 6.55/10^5^; rectum cancer in women:2.72/10^5^, respectively) were well above the global average in 2020 (colon cancer in men: 6.4/10^5^; colon cancer in women: 4.6/10^5^; rectum cancer in men: 4.4/10^5^; rectum cancer in women: 2.4/10^5^, respectively) and similar to the European levels (colon cancer in men: 9.8/10^5^; colon cancer in women: 6.2/10^5^; rectum cancer in men: 6.1/10^5^; rectum cancer in women: 3.1/10^5^, respectively).

We did not observe remarkable trends of ASIRs and ASMRs for cancers of the colon and rectum from 2010 to 2019. However, both the number of cases and crude rates consistently increased yearly from 1975–1979 to 2015–2019 [34]. The difference between the crude rates and the age-standardized rate is the large proportion of the elderly population in Shanghai. The rapid development of the economy and society likely reflects changes in lifestyle factors, obesity, physical activity and diet, that is, shifts toward an increased intake of animal-source foods and a more sedentary lifestyle, resulting in reduced physical activity and increased prevalence of excess body weight, which are independently associated with colorectal cancer risk [35, 36]. Additional major risk factors included heavy alcohol consumption, cigarette smoking, and consumption of red or processed meat [34, 37–39]. The city government of Shanghai has launched a community-based colorectal cancer screening program since 2013, which may be related to an increase in the number of cases of colorectal cancer [40]. However, it has not affected the overall trends of ASIRs and has not fully reflected the effect of reducing ASMRs.

The ASIRs of liver cancer in the study area from 2015 to 2019 were 13.78/10^5^ in men and 4.33/10^5^ in women, slightly lower than the global average (men: 14.1/10^5^; women: 5.2/10^5^) and about half of the China average (men: 27.6/10^5^; women: 9.0/10^5^) [1, 33]. The results of the ASMRs and ASIRs were similar, reflecting a bad prognosis of liver cancer in the district. Significantly downward trends were observed in the ASIRs and ASMRs of liver cancer between 2010 and 2019, consistent with our previous finding [41]. While a relatively stable crude rate of liver cancer suggests that liver cancer is still a challenge in the future. The main risk factors for liver cancer are chronic infection with hepatitis B virus (HBV) or hepatitis C virus (HCV), aflatoxin-contaminated foods, heavy alcohol intake, excess body weight, type 2 diabetes, and smoking [42–44]. Since 1992, the Chinese government has mandated HBV vaccinations for newborns, and it began to see a large reduction in the numbers of HBV infections in mainland China. Moreover, relative to the rest of China, higher economic and living standards in urban Shanghai, together with high-quality medical and health resources, might contribute to the decreased rate of liver cancer.

The lifetime risk of developing cancer is the percentage of the population developing cancer at least once in a lifetime [16]. Age-conditional risk of developing cancer is the percentage of the population developing cancer before a specific age, assuming that the individuals are cancer-free at the current age [16]. Lifetime and age-conditional risks of dying from cancer are defined in the same manner as the risks of developing cancer. These indicators are helpful for planning, monitoring and evaluating cancer control programs. However, there is no literature on the lifetime risk analysis of cancer in China at present. The lifetime risks of developing and dying from digestive tract cancers in the Changning District of Shanghai from 2010 to 2019 were significantly higher than that in the United States from 2016 to 2018. For example, the lifetime risks of developing liver cancer in our study were 2.76% in men and 1.19% in women, while figures in the United States from 2016 to 2018 were 1.45% in men and 0.64% in women [45]. Lifetime or age-conditional risk is a very familiar index for nations because the risk of cancer can be converted to a percentage for each situation. Especially, the interpretation using the reciprocal number of this probability, for example, one in twenty-one males will develop stomach cancer and one in twenty-seven males will die of stomach cancer during their lifetime, makes the risk for cancer an intuitively comprehensible form [46].

The age-specific incidence and mortality rates of digestive tract cancers increased gradually with age, especially rapidly increasing after 50 years, and reaching a peak in people over 80 years old. Our study shows that most incidence cases and deaths of digestive tract cancers have occurred after 50 years. The changing trends of incidence or mortality are different in different age groups. For example, we found that rectum cancer incidence in the age group of 55–64 increased significantly in men, which was the same as the previous study in Shanghai [47]. As in previous studies [26, 35, 48], this may partly reflect the effect of screening. Screening is one of the effective means of cancer prevention [26]. With the gradual popularization of screening in China [49], its effect on digestive tract cancer is also worthy of attention.

Our study also has several limitations. First, the findings of this study may not be representative of other Chinese populations, especially in rural areas. Second, due to the lack of demographic information over the age of 85, we can only estimate the probability of developing and dying from cancer from birth to the age of 85. Thirdly, exposures to risk factors including socioeconomic status, smoking, alcohol use, obesity, physical activity, diet habit, and chronic infection with HBV and HCV are important to characterize the controllable causes of cancer types in different areas. However, these data were unavailable. But our study results provided informative statistics for the local administration section of public health.

## Conclusion

In conclusion, the burden of digestive tract cancer and its disparities between sex and age group remain major public health challenges in urban Shanghai. With the huge population size and the continuity of aging, attention should also be paid to digestive tract cancers for a long time. Therefore, government and researchers should develop and promote a healthy diet, organize a screening, and reduce the prevalence of smoking, alcohol drinking, and hepatitis B virus and hepatitis C virus infections, to reduce the burden of digestive tract cancers.

## Supplementary Information

Below is the link to the electronic supplementary material.

Supplementary file1 (DOCX 24 KB)

## Data Availability

The data will be available on request pending approval by the scientific committee of the relevant institutes.

## Abbreviations

NCC: National Cancer Center
ASR: Age-standardized rate
ASIR: Age-standardized incidence rate
ASMR: Age-standardized mortality rate
ASIRs: Age-standardized incidence rates
ASMRs: Age-standardized mortality rates
ICD-10: International Classification of Diseases, the tenth revision
M/I: Mortality to incidence ratio
HV: Histologically verified
DCO: Death certification only
APC: The annual percentage change
HBV: Hepatitis B virus
HCV: Hepatitis C virus
IARC: International Agency for Research on Cancer

## References

[1] Sung H, Ferlay J, Siegel RL, Laversanne M, Soerjomataram I, Jemal A (2021). Global cancer statistics 2020: GLOBOCAN estimates of incidence and mortality worldwide for 36 cancers in 185 countries. CA Cancer J Clin.

[2] Chen W, Zheng R, Baade PD, Zhang S, Zeng H, Bray F (2016). Cancer statistics in China, 2015. CA Cancer J Clin.

[3] Zheng R, Zhang S, Zeng H, Wang S, Sun K, Chen R (2022). Cancer incidence and mortality in China, 2016. J Natl Cancer Center.

[4] Jiang YF, Li ZY, Ji XW, Shen QM, Tuo JY, Yuan HY (2020). Global pattern and trend of liver cancer survival: a systematic review of population-based studies. Hepatoma Res.

[5] Chen W, Xia C, Zheng R, Zhou M, Lin C, Zeng H (2019). Disparities by province, age, and sex in site-specific cancer burden attributable to 23 potentially modifiable risk factors in China: a comparative risk assessment. Lancet Glob Health.

[6] Wu Y, Li Y, Giovannucci E (2021). Potential impact of time trend of lifestyle risk factors on burden of major gastrointestinal cancers in China. Gastroenterology.

[7] Shao Y, Hua Z, Zhao L, Shen Y, Guo X, Niu C (2018). Time trends of gastrointestinal cancers incidence and mortality in Yangzhong from 1991 to 2015: an updated age-period-cohort analysis. Front Oncol.

[8] Li X, Deng Y, Tang W, Sun Q, Chen Y, Yang C (2018). Urban-rural disparity in cancer incidence, mortality, and survivals in Shanghai, China, during 2002 and 2015. Front Oncol.

[9] Kerr J, Anderson C, Lippman SM (2017). Physical activity, sedentary behaviour, diet, and cancer: an update and emerging new evidence. Lancet Oncol.

[10] Yang WS, Zeng XF, Liu ZN, Zhao QH, Tan YT, Gao J (2020). Diet and liver cancer risk: a narrative review of epidemiological evidence. Br J Nutr.

[11] Bray F, Colombet M, Mery L, Piñeros M, Znaor A, Zanetti R (2017). Cancer incidence in five continents.

[12] Bray F, Ferlay J, Laversanne M, Brewster D, Gombe Mbalawa C, Kohler B (2015). Cancer incidence in five continents: inclusion criteria, highlights from Volume X and the global status of cancer registration. Int J Cancer.

[13] Gao YT, Lu W (2007). Cancer incidence, mortality and survival rates in urban Shanghai (1973–2000).

[14] Center NC (2016). Chinese guideline for cancer registration.

[15] Jensen OM, Parkin DM, MacLennan R, Muir CS, Skeet R (1991). Cancer registration: principles and methods.

[16] Fay MP, Pfeiffer R, Cronin KA, Le C, Feuer EJ (2003). Age-conditional probabilities of developing cancer. Stat Med.

[17] Fay MP (2004). Estimating age conditional probability of developing disease from surveillance data. Popul Health Metr.

[18] Kim HJ, Fay MP, Feuer EJ, Midthune DN (2000). Permutation tests for joinpoint regression with applications to cancer rates. Stat Med.

[19] Michelle Dunn JZ. AAPC for the joinpoint connect-the-dots scenario, 2009–02. Statistical Research and Applications Branch, National Cancer Institute; 2009. https://surveillance.cancer.gov/reports. Accessed 1 Jan 2022.

[20] Zeng H, Chen W, Zheng R, Zhang S, Ji JS, Zou X (2018). Changing cancer survival in China during 2003–15: a pooled analysis of 17 population-based cancer registries. Lancet Glob Health.

[21] den Hoed CM, Kuipers EJ (2016). Gastric cancer: how can we reduce the incidence of this disease?. Curr Gastroenterol Rep.

[22] Karimi P, Islami F, Anandasabapathy S, Freedman ND, Kamangar F (2014). Gastric cancer: descriptive epidemiology, risk factors, screening, and prevention. Cancer Epidemiol Biomark Prev.

[23] Cao MM, Li H, Sun DQ, He SY, Lei L (2021). Epidemiological trend analysis of gastric cancer in China from 2000 to 2019. Chin J Dig Surg.

[24] Fang J, Jiang Y, Li HL, Zhou P, Zhang W, Zhang L (2019). Time trends of gastric cancer incidence and mortality in Changning district of Shanghai, 1988–2013. China Cancer.

[25] GBD 2017 Stomach Cancer Collaborators. The global, regional, and national burden of stomach cancer in 195 countries, 1990–2017: a systematic analysis for the Global Burden of Disease study 2017. Lancet Gastroenterol Hepatol. 2020;5(1):42–54. 10.1016/S2468-1253(19)30328-0 PMC7033564 31648970

[26] Thrift AP, El-Serag HB (2020). Burden of gastric cancer. Clin Gastroenterol Hepatol.

[27] Yang L, Zheng RS, Wang N, Yuan NN, Liu S, Li HC (2018). Incidence and mortality of stomach cancer in China, 2014. Chin J Cancer Res.

[28] Zha ZQ, Li R, Hu MJ, Dai D, Song L, Huang F (2020). Analysis on the relationship between smoking status and the onset age of onset and the direct medical expenditure expenses of gastric cancer patients. Chin J Epidemiol.

[29] Millwood IY, Li L, Smith M, Guo Y, Yang L, Bian Z (2017). Alcohol consumption in 0.5 million people from 10 diverse regions of China: prevalence, patterns and socio-demographic and health-related correlates. Int J Epidemiol.

[30] Deng W, Jin L, Zhuo H, Vasiliou V, Zhang Y (2021). Alcohol consumption and risk of stomach cancer: a meta-analysis. Chem Biol Interact.

[31] Fidler MM, Soerjomataram I, Bray F (2016). A global view on cancer incidence and national levels of the human development index. Int J Cancer.

[32] Fidler MM SI, Bray F. Transitions in human development and the global cancer burden. World Cancer Report 2014. WHO Press; 2014. p. 42–55.

[33] Ferlay J EM, Lam F, Colombet M, Mery L, Piñeros M, Znaor A, et al. Global cancer observatory: cancer today. Lyon: International Agency for Research on Cancer; 2020. https://gco.iarc.fr/today. Accessed 14 Dec 2021.

[34] Wu H, Zhou P, Zhang W, Jiang Y, Liu XL, Zhang L (2018). Time trends of incidence and mortality in colorectal cancer in Changning District, Shanghai, 1975–2013. J Dig Dis.

[35] Siegel RL, Miller KD, Goding Sauer A, Fedewa SA, Butterly LF, Anderson JC (2020). Colorectal cancer statistics, 2020. CA Cancer J Clin.

[36] Thanikachalam K, Khan G (2019). Colorectal cancer and nutrition. Nutrients.

[37] World Cancer Research Fund/American Institute for Cancer Research. The Continuous Update Project Expert Report 2018. Diet, Nutrition, Physical Activity and Cancer: Colorectal Cancer. 2018. https://wcrf.org/sites/default/files/Colorectal-cancer-report.pdf. Accessed 1 Jan 2022.

[38] Hull MA, Rees CJ, Sharp L, Koo S (2020). A risk-stratified approach to colorectal cancer prevention and diagnosis. Nat Rev Gastroenterol Hepatol.

[39] Cho YA, Lee J, Oh JH, Chang HJ, Sohn DK, Shin A (2019). Genetic risk score, sombined lifestyle Factors and risk of colorectal cancer. Cancer Res Treat.

[40] Gong Y, Peng P, Bao P, Zhong W, Shi Y, Gu K (2018). The implementation and first-round results of a community-based colorectal cancer screening program in Shanghai, China. Oncologist.

[41] Ji XW, Jiang Y, Wu H, Zhou P, Tan YT, Li HL (2020). Long-term liver cancer incidence and mortality trends in the Changning District of Shanghai, China. J Dig Dis.

[42] Thomas LW, Petrick JL, McGlynn KA. Cancer epidemiology and prevention. Liver cancer. 4th ed. Oxford University Press; 2018.

[43] Petrick JL, Yang B, Altekruse SF, Van Dyke AL, Koshiol J, Graubard BI (2017). Risk factors for intrahepatic and extrahepatic cholangiocarcinoma in the United States: a population-based study in SEER-Medicare. PLoS ONE.

[44] Chimed T, Sandagdorj T, Znaor A, Laversanne M, Tseveen B, Genden P (2017). Cancer incidence and cancer control in Mongolia: results from the National Cancer Registry 2008–12. Int J Cancer.

[45] Howlader N, Noone AM, Krapcho M, Miller D, Brest A, Yu M, et al. SEER cancer statistics review, 1975–2018. https://seer.cancer.gov/csr/1975_2018. Accessed 1 Jan 2022.

[46] Kamo K, Katanoda K, Matsuda T, Marugame T, Ajiki W, Sobue T (2008). Lifetime and age-conditional probabilities of developing or dying of cancer in Japan. Jpn J Clin Oncol.

[47] Yan YJ, Zhang F, Li WX, Zhou J, Xu HL, Fang H (2016). Temporal trend and features of colorectal cancer incidence in Minhang district of Shanghai, 2002–2012. J Environ Occup Med.

[48] Fedewa SA, Siegel RL, Jemal A (2019). Are temporal trends in colonoscopy among young adults concordant with colorectal cancer incidence?. J Med Screen.

[49] Chen HD, Dai M (2020). On prevention and control strategy of colorectal cancer in China. Chin J Epidemiol.

