# Supplementary material for: Incidence, Mortality Features and Lifetime Risk Estimation of Digestive Tract Cancers in an Urban District of Shanghai, China
Source: J Epidemiol Glob Health. 2022 Jun 25;12(3):248–57. doi: 10.1007/s44197-022-00047-3 (PMC9470802; doi:10.1007/s44197-022-00047-3)
Supplement: Supplementary file 1 — Supplementary file1 (DOCX 24 KB) [file 44197_2022_47_MOESM1_ESM.docx]

Supplementary Table 1 Data quality indices for digestive tract cancers in the Changning district, Shanghai, China, 2010-2019

| Types | Basis of diagnosis with highest reliability | | | | | | | M/I | |
| --- | --- | --- | --- | --- | --- | --- | --- | --- | --- |
|  | HV | Biochemistry | Surgery | Imaging | Clinical | DCO |  | |  |
| Stomach | 78.74 | 5.09 | 6.54 | 7.24 | 2.35 | 0.04 | 0.73 | |  |
| Colon | 81.18 | 4.48 | 6.40 | 6.09 | 1.78 | 0.07 | 0.56 | |  |
| Rectum | 86.65 | 3.26 | 4.31 | 3.45 | 2.28 | 0.06 | 0.58 | |  |
| Liver | 31.69 | 24.84 | 16.34 | 22.53 | 4.48 | 0.13 | 0.88 | |  |
| All | 72.77 | 8.02 | 7.80 | 8.83 | 2.52 | 0.07 | 0.67 | |  |

HV: histologically verified percentage;

DCO: death certification only;

M/I:mortality/incidence.

Supplementary Table 2 Joinpoint regression analysis of incidence for digestive tract cancers in men by age in the Changning District, Shanghai, China, 2010-2019.

| Sites | AAPC (%, 95%CI) | Trend 1 |  | Trend 2 |  | Trend 3 |  |
| --- | --- | --- | --- | --- | --- | --- | --- |
|  |  | Years | APC (%, 95% CI) | Years | APC (%, 95% CI) | Years | APC (%, 95% CI) |
| Stomach |  |  |  |  |  |  |  |
| 0-44 | 6.88 (-3.59, 18.47) | 2010-2019 | 6.88 (-3.59, 18.47) |  |  |  |  |
| 45-54 | -2.78 (-9.6, 4.55) | 2010-2019 | -2.78 (-9.6, 4.55) |  |  |  |  |
| 55-64 | 0.58 (-3.33, 4.65) | 2010-2019 | 0.58 (-3.33, 4.65) |  |  |  |  |
| 65-74 | -2.38 (-7.61, 3.15) | 2010-2019 | -2.38 (-7.61, 3.15) |  |  |  |  |
| 75+ | -2.25 (-5.55, 1.17) | 2010-2019 | -2.25 (-5.55, 1.17) |  |  |  |  |
| Colon |  |  |  |  |  |  |  |
| 0-44 | - |  |  |  |  |  |  |
| 45-54 | 3.8 (-0.6, 8.4) | 2010-2019 | 3.8 (-0.6, 8.4) |  |  |  |  |
| 55-64 | 6.24 (-0.43, 13.36) | 2010-2019 | 6.24 (-0.43, 13.36) |  |  |  |  |
| 65-74 | -2.89 (-8.73, 3.32) | 2010-2019 | -2.89 (-8.73, 3.32) |  |  |  |  |
| 75+ | 3.87 (-0.98, 8.96) | 2010-2012 | 34.65* (4.27, 73.88) | 2012-2019 | -3.55* (-6.79, -0.2) |  |  |
| Rectum |  |  |  |  |  |  |  |
| 0-44 | 16.33* (1.41, 33.44) | 2010-2019 | 16.33* (1.41, 33.44) |  |  |  |  |
| 45-54 | 5.44 (-16.71, 33.47) | 2010-2013 | 22.73 (-40.7, 154.03) | 2013-2017 | -21.23 (-61.94, 63.05) | 2017-2019 | 50.4(-64.89,544.31) |
| 55-64 | 10.36* (0.02, 21.76) | 2010-2012 | 55.54 (-8.06, 163.16) | 2012-2019 | 0.05 (-6.74, 7.33) |  |  |
| 65-74 | -2.37 (-8.44, 4.1) | 2010-2019 | -2.37 (-8.44, 4.1) |  |  |  |  |
| 75+ | -0.46 (-6.34, 5.78) | 2010-2019 | -0.46 (-6.34, 5.78) |  |  |  |  |
| Liver |  |  |  |  |  |  |  |
| 0-44 | -8.67 (-20.74, 5.24) | 2010-2019 | -8.67 (-20.74, 5.24) |  |  |  |  |
| 45-54 | -7.22* (-11.94, -2.24) | 2010-2019 | -7.22* (-11.94, -2.24) |  |  |  |  |
| 55-64 | -7.00* (-12.97, -0.62) | 2010-2019 | -7.00* (-12.97, -0.62) |  |  |  |  |
| 65-74 | -5.4 (-12.28, 2.02) | 2010-2019 | -5.4 (-12.28, 2.02) |  |  |  |  |
| 75+ | 1.21 (-3.3, 5.93) | 2010-2019 | 1.21 (-3.3, 5.93) |  |  |  |  |

AAPC: average annual percent change; APC: annual percent change; CI: confidence interval; *:P<0.05; -: lack of data.

.

Supplementary Table 3 Joinpoint regression analysis of incidence for digestive tract cancers in women by age in the Changning District, Shanghai, China, 2010-2019.

| Sites | AAPC (%, 95%CI) | Trend 1 |  |
| --- | --- | --- | --- |
|  |  | Years | APC (%, 95%CI) |
| Stomach |  |  |  |
| 0-44 | -0.44 (-13.33, 14.36) | 2010-2019 | -0.44 (-13.33, 14.36) |
| 45-54 | -5.46 (-18.18, 9.25) | 2010-2019 | -5.46 (-18.18, 9.25) |
| 55-64 | -0.5 (-4.84, 4.04) | 2010-2019 | -0.5 (-4.84, 4.04) |
| 65-74 | -2.62 (-8.98, 4.18) | 2010-2019 | -2.62 (-8.98, 4.18) |
| 75+ | 0.07 (-2.98, 3.2) | 2010-2019 | 0.07 (-2.98, 3.2) |
| Colon |  |  |  |
| 0-44 | 7.8 (-4.72, 21.96) | 2010-2019 | 7.8 (-4.72, 21.96) |
| 45-54 | -0.65 (-10.89, 10.76) | 2010-2019 | -0.65 (-10.89, 10.76) |
| 55-64 | 2.16 (-2.72, 7.29) | 2010-2019 | 2.16 (-2.72, 7.29) |
| 65-74 | -5.62* (-10.19, -0.82) | 2010-2019 | -5.62* (-10.19, -0.82) |
| 75+ | -0.72 (-4.75, 3.47) | 2010-2019 | -0.72 (-4.75, 3.47) |
| Rectum |  |  |  |
| 0-44 | 5.51 (-7.48, 20.32) | 2010-2019 | 5.51 (-7.48, 20.32) |
| 45-54 | -3.74 (-15.51, 9.67) | 2010-2019 | -3.74 (-15.51, 9.67) |
| 55-64 | -1.71 (-7.64, 4.59) | 2010-2019 | -1.71 (-7.64, 4.59) |
| 65-74 | -7.52* (-13.32, -1.34) | 2010-2019 | -7.52* (-13.32, -1.34) |
| 75+ | 0.49 (-5.91, 7.33) | 2010-2019 | 0.49 (-5.91, 7.33) |
| Liver |  |  |  |
| 0-44 | - |  |  |
| 45-54 | - |  |  |
| 55-64 | -4.52 (-10.29, 1.62) | 2010-2019 | -4.52 (-10.29, 1.62) |
| 65-74 | -5.47 (-13.56, 3.38) | 2010-2019 | -5.47 (-13.56, 3.38) |
| 75+ | -1.9 (-7.03, 3.51) | 2010-2019 | -1.9 (-7.03, 3.51) |

AAPC: average annual percent change; APC: annual percent change; CI: confidence interval; *:P<0.05; -: lack of data.

Supplementary Table 4 Joinpoint regression analysis of mortality for digestive tract cancers in women by age in the Changning District, Shanghai, China, 2010-2019.

| Sitess | AAPC (%, 95%CI) | Trend 1 |  |
| --- | --- | --- | --- |
|  |  | Years | APC (%, 95%CI) |
| Stomach |  |  |  |
| 0-44 | - |  |  |
| 45-54 | -7.54* (-14.34, -0.19) | 2010-2019 | -7.54* (-14.34, -0.19) |
| 55-64 | -2.73 (-10.49, 5.7) | 2010-2019 | -2.73 (-10.49, 5.7) |
| 65-74 | -8.07* (-11, -5.04) | 2010-2012 | -8.07* (-11, -5.04) |
| 75+ | -0.98 (-4.31, 2.46) | 2010-2019 | -0.98 (-4.31, 2.46) |
| Colon |  |  |  |
| 0-44 | - |  |  |
| 45-54 | -2.37 (-15.08, 12.24) | 2010-2019 | -2.37 (-15.08, 12.24) |
| 55-64 | 2.44 (-2.9, 8.07) | 2010-2019 | 2.44 (-2.9, 8.07) |
| 65-74 | -1.05 (-7.33, 5.65) | 2010-2019 | -1.05 (-7.33, 5.65) |
| 75+ | 4.35 (-0.68, 9.65) | 2010-2019 | 4.35 (-0.68, 9.65) |
| Rectum |  |  |  |
| 0-44 | - |  |  |
| 45-54 | -4.06 (-15.72, 9.21) | 2010-2019 | -4.06 (-15.72, 9.21) |
| 55-64 | 8.56 (-0.58, 18.55) | 2010-20121 | 8.56 (-0.58, 18.55) |
| 65-74 | 2.47 (-6.57, 12.38) | 2010-2019 | 2.47 (-6.57, 12.38) |
| 75+ | 1.3 (-2.58, 5.33) | 2010-2019 | 1.3 (-2.58, 5.33) |
| Liver |  |  |  |
| 0-44 | -7.82 (-19.85, 6.01) | 2010-2019 | -7.82 (-19.85, 6.01) |
| 45-54 | -6.87 (-17.51, 5.14) | 2010-2019 | -6.87 (-17.51, 5.14) |
| 55-64 | -6.61* (-11.27, -1.71) | 2010-2019 | -6.61* (-11.27, -1.71) |
| 65-74 | -6.22* (-11.73, -0.38) | 2010-2019 | -6.22* (-11.73, -0.38) |
| 75+ | -1.5 (-5.16, 2.29) | 2010-2019 | -1.5 (-5.16, 2.29) |

AAPC: average annual percent change; APC: annual percent change; CI: confidence interval; *:P<0.05; -: lack of data.

Supplementary Table 5 Joinpoint regression analysis of mortality for digestive tract cancers in women by age in the Changning District, Shanghai, China, 2010-2019.

| Sites | AAPC (%, 95%CI) | Trend 1 |  |
| --- | --- | --- | --- |
|  |  | Years | APC (%, 95%CI) |
| Stomach |  |  |  |
| 0-44 | - |  |  |
| 45-54 | - |  |  |
| 55-64 | -4.15 (-13.3, 5.96) | 2010-2019 | -4.15 (-13.3, 5.96) |
| 65-74 | -7.60* (-14.59, -0.04) | 2010-2019 | -7.60* (-14.59, -0.04) |
| 75+ | -0.93 (-4.98, 3.28) | 2010-2019 | -0.93 (-4.98, 3.28) |
| Colon |  |  |  |
| 0-44 | - |  |  |
| 45-54 | - |  |  |
| 55-64 | 4.57 (-1.06, 10.52) | 2010-2019 | 4.57 (-1.06, 10.52) |
| 65-74 | -8.66 (-16.9, 0.4) | 2010-2019 | -8.66 (-16.9, 0.4) |
| 75+ | 2.51 (-0.04, 5.13) | 2010-20121 | 2.51 (-0.04, 5.13) |
| Rectum |  |  |  |
| 0-44 | - |  |  |
| 45-54 | - |  |  |
| 55-64 | -12.14 (-23.72, 1.21) | 2010-2012 | -12.14 (-23.72, 1.21) |
| 65-74 | - |  |  |
| 75+ | 4.44* (0.79, 8.22) | 2010-2019 | 4.44* (0.79, 8.22) |
| Liver |  |  |  |
| 0-44 | - |  |  |
| 45-54 | - |  |  |
| 55-64 | -9.6 (-18.91, 0.77) | 2010-2019 | -9.6 (-18.91, 0.77) |
| 65-74 | -4.97 (-17.17, 9.04) | 2010-2019 | -4.97 (-17.17, 9.04) |
| 75+ | 2.47 (-4.32, 9.74) | 2010-2019 | 2.47 (-4.32, 9.74) |

AAPC: average annual percent change; APC: annual percent change; CI: confidence interval; *:P<0.05; -: lack of data.
